# SNP markers for low molecular glutenin subunits (LMW-GSs) at the *Glu-A3* and *Glu-B3* loci in bread wheat

**DOI:** 10.1371/journal.pone.0233056

**Published:** 2020-05-12

**Authors:** Susanne Dreisigacker, Yonggui Xiao, Deepmala Sehgal, Carlos Guzman, Zhonghu He, Xianchun Xia, Roberto J. Peña

**Affiliations:** 1 Global Wheat Program, International Maize and Wheat Improvement Center (CIMMYT), Texcoco, Mexico; 2 Institute of Crop Sciences, National Wheat Improvement Center, Chinese Academy of Agricultural Sciences (CAAS), Beijing, China; 3 Departamento de Genética, Escuela Técnica Superior de Ingeniería Agronómica y de Montes, Universidad de Córdoba, Córdoba, Spain; 4 International Maize and Wheat Improvement Center (CIMMYT) China Office, Beijing, China; Murdoch University, AUSTRALIA

## Abstract

The content and composition of seed storage proteins is largely responsible for wheat end-use quality. They mainly consist of polymeric glutenins and monomeric gliadins. According to their electrophoretic mobility, gliadins and glutenins are subdivided into several fractions. Glutenins are classified as high molecular weight or low molecular weight glutenin subunits (HMW-GSs and LMW-GSs, respectively). LMW-GSs are encoded by multigene families located at the orthologous *Glu-3* loci. We designed a set of 16 single-nucleotide polymorphism (SNP) markers that are able to detect SDS-PAGE alleles at the *Glu-A3* and *Glu-B3* loci. The SNP markers captured the diversity of alleles in 88 international reference lines and 27 Mexican cultivars, when compared to SDS-PAGE and STS markers, however, showed a slightly larger percent of multiple alleles, mainly for *Glu-B3*. SNP markers were then used to determine the *Glu-1* and *Glu-3* allele composition in 54 CIMMYT historical lines and demonstrated to be useful tool for breeding programs to improve wheat end product properties.

## Introduction

The world population is growing exponentially and therefore demands more and a greater diversity of food while facing less available land and the need to conserve soil, water, and genetic resources. More than any other crop, wheat (*Triticum aestivum* L.) provides calories and protein and is present in thousands of everyday foods worldwide. The global bread wheat consumption supplies nearly 16 g of protein per capita daily and is quickly increasing in developing countries, which are predicted to have the largest population increases [1].

The major problem is that even though wheat yields are increasing [2,3], the percentage increase is below the projected percentage demand with about 0.6% deficit projected annually until 2050 [4,5]. And while overall production must increase, high-quality standards for human nutrition, end-use functional properties and commodity value must also be maintained.

Wheat processing quality and end product properties are determined by a set of complex traits, the most important being the content and composition of seed storage proteins (gluten), the endosperm texture or grain hardness, the composition of starch and non-starch polysaccharides, and, for some specific products, the color of the flour/semolina. Gluten proteins, representing the major protein fraction of the starchy endosperm, are the main factors responsible for the unique viscoelastic properties (elasticity or strength and extensibility) of wheat dough, which underline the utilization of wheat to prepare bread and other wheat products [6,7]. Gluten is composed of a large number of proteins, mainly glutenins and gliadins. Glutenins contribute more to gluten strength while gliadins contribute to extensibility and viscosity. Among the glutenins there are high-molecular-weight glutenin subunits (HMW-GS), and low-molecular-weight glutenin subunits (LMW-GS). Common wheat possesses three to five HMW-GS codified by the *Glu-A1*, *Glu-B1* and *Glu-D1* loci and encoded on the long arm of chromosomes 1A, 1B and 1D, respectively. Although HMW-GS account for only 10% of the wheat storage proteins, they play a key role in determining bread wheat quality. The LMW-GS are codified by the *Glu-A3*, *Glu-B3* and *Glu-D3* loci (located on the short arms of chromosomes 1A, 1B and 1D, respectively) and large allelic variation has been reported with different alleles being designated by the name of the locus followed by letters, [8,9]. The LMW-GS represent about one-third of the total seed protein and around 60% of total glutenins [10]. Therefore, the characterization of allelic variation for LMW-GS in addition to HMW-GS among cultivars and investigation of their relationships with end-use quality has been a key area of research on quality improvement [11]. The LMW-GS have, however, proven to be more difficult to identify because of their complexity, heterogeneity, and similarity to each other and to some gliadin components.

To ensure good quality products, wheat quality analyses are an integral part of most breeding programs. Typically, parental lines for new crosses and advanced lines are evaluated. Quality analyses are rarely conducted during the early breeding generation selection process, mainly because the screening of entries in heterozygous state within segregating populations is not indicative in comparison to pure lines, early testing for quality must not compromise other breeding priorities, which must run with finite testing resources and high throughput, accurate methodologies are not available to analyze the extremely high number of entries present in early generations.

Single nucleotide polymorphisms (SNPs) and insertion/deletions (InDels) are common genetic polymorphisms within the HMW-GS and LMW-GS genes. PCR based sequence-tagged site (STS) markers exist for ascertaining most HMW-GS [12–15]. Recently, LMW-GS genes were also isolated and functional markers identifying the subunit composition on chromosomes 1A and 1B in common wheat were developed to allow marker-assisted breeding [16,17]. However, despite being closely linked or functional markers, the application of these remains limited largely due to their dominant inheritance coupled with the cost and time required for screening large progenies via conventional PCR and gel electrophoresis.

The objective of our study was therefore to develop and validate a set of SNP markers functional for *Glu-A3* and *Glu-B3* alleles and useful for screening large number of samples in marker-assisted breeding. SNP marker results were validated by full comparison to sodiumdodecyl-sulfate polyacrylamide gel electrophoresis (SDS-PAGE) and STS marker methods.

## Materials and methods

### Plant materials

Two germplasm sets were used to validate the designed SNP markers for *Glu-A3* and *Glu-B3* alleles. The first set (Set1) comprised 88 international reference lines with maximized diversity for *Glu-A3* and *Glu-B3* alleles (Table 1). The set was developed by the Expert Working Group on Improving Wheat Quality for Processing and Health of the Wheat Initiative (https://www.wheatinitiative.org/). Seed was compiled from different sources and multiplied in Mexicali, México, and is available for distribution through the CIMMYT Germplasm Bank.

**Table 1:** Compositions of LMW-GS alleles in 88 reference lines used to establish a uniform classification system identified by SDS-PAGE, allele specific STS markers and allele specific KASP assays.

No.	GID	Specific Name	Country	Glu-A3^a^	Glu-B3 ^a^
1	7810950	ACA 303	ARGENTINA	f/f/f	h/h/h
2	7810951	CHINESE SPRING	CHINA	a/a/a	a/a/a
3	7810952	HALBERD	AUSTRALIA	e/e/e	c'/c/c
4	7810953	NORIN 61	JAPAN	d/d/d	i/f or g/f
5	7810954	GREEBE	AUSTRALIA	c/e/e	j/_/_
6	7810955	FESTIN	FRANCE	f/f/_	b/b/b
7	7810956	SOISSONS	ITALY	c/c/c	b'/b/b
8	7810957	PETREL	FRANCE	d/d/d	h/h/h
9	7810958	BRIMSTONE	UNITED KINGDOM	c/c/c	g/_/c or g
10	7810959	MAGALI-BLONDEAU	FRANCE	e/e/e	f/f/f
11	7810960	PEPITAL	NETHERLANDS	f/f/f	d/d/d
12	7810961	CAPPELLE DESPREZ	FRANCE	d/c/c	g/g or i/c or g or i
13	7810962	THESEE	FRANCE	c/c/c	g/h/h
14	7810963	JING 411	CHINA	c/d/d	h/d/h
15	7810964	ORCA	NETHERLANDS	d/d/d	d/g/c or g
16	7810965	COURTOT	FRANCE	c/c/c	b/f/f
17	7810966	ETOILE DE CHOISY	FRANCE	d/d or f/d	i/i/i
18	7810967	NANBU KOMUGI	JAPAN	d/d or f/d	b'/b/b
19	7810968	SHINCHUNAGA	JAPAN	c/c/c	i/f or g/f
20	7810969	CLEMENT	NETHERLANDS	f/f/f	j/_/_
21	7810970	99G46	CHINA	f/f/f	j/_/_
22	7810971	ACA 601	ARGENTINA	f/e or f/_	b'/b/b
23	7810972	ACA 801	ARGENTINA	c/c/c	g/g or i/c or g or i
24	7810973	AOBA KOMUGI	JAPAN	e/e/e	b/b/b
25	7810974	APOLLO	GERMANY	d/d/d	j/_/_
26	7810975	BUCK BRASIL	ARGENTINA	f/f/f	g/g or i/c or g
27	7810976	BUCK MEJORPAN	ARGENTINA	f/f/f	b'/b/b
28	7810977	BUCK PINGO	ARGENTINA	f/f/f	i/i/i
29	7810978	CA9641	CHINA	d/d/d	h/h/h
30	7810979	CHOPIN	FRANCE	c/c/_	h/_/_
31	7812093	ESCHIMA/SHINRIKI	JAPAN	c/c/c	d/d/d
32	7810980	GUANFENG 2	CHINA	c/c/c	b/b/b
33	7810981	HARUYUTAKA		c/c/c	h/g or h/h
34	7810982	KITANOKAORI	JAPAN	f/f/f	j/_/_
35	7810983	KLEIN CAPRICORNIO	ARGENTINA	c/c/c	h/h/h
36	7810984	KLEIN JABALI	ARGENTINA	g/g/_	f or g /g/c or g
37	7810985	KLEIN PROTEO	ARGENTINA	g/c/_	g/_/_
38	7810986	LUMAI 23	CHINA	c/c/c	d/d/d
39	7810987	MAGDALENA	FRANCE	d/d/d	b'/b/b
40	7810988	MANITAL	ITALY	c/c/c	b/b/b
41	7810989	NEIXIANG 188	CHINA	a/a or f/a	j/_/_
42	7810990	NIDERA BAGUETTE 10	ARGENTINA	d/c or d/d	g/g or b/c or g
43	7810991	NIDERA BAGUETTE 20	ARGENTINA	f/c/f	f or g /g/c or g
44	7810992	NORIN 67	JAPAN	c/c/c	f or g /g/c or g
45	7810993	PROINTA AMANECER	ARGENTINA	f/_/f	j/_/_
46	7810994	PROINTA COLIBRI	ARGENTINA	d/d/d	b/b/b
47	7810995	PROINTA ISLA VERDE	ARGENTINA	b/b/b	b/b/b
48	7810996	PROINTA REDOMON	ARGENTINA	c/c/c	h/h/h
49	7810997	RENAN	ARGENTINA	f/f/f	b/b/b
50	7810998	RUSO	ARGENTINA	c/c/c	i/i/i
51	7810999	SALOMNE	ITALY	c/c/c	c/g/c or g
52	7811000	SHAN 229	CHINA	c/c/c	j/_/_
53	7812057	SHIRANEKOMUGI	JAPAN	e/e/e	i/i/i
54	7812058	THOMAS NEVADO	ARGENTINA	c/c/c	j/_/_
55	7812059	YANGMAI 158	CHINA	c/c/c	f or g/g/c or g
56	7812060	YUMAI 54	CHINA	c/c/c	d/d/d
57	7812061	YUMAI 69	CHINA	c/c/c	d/d/d
58	7812062	ZHONGYOU 9507	CHINA	d/d/d	b/b/b
59	7812063	ZHONGYOU 9701	CHINA	d/d/d	d/d/d
60	7812064	ZHONGYU 415	CHINA	c/c/c	d/d/d
61	7812065	AMADINA	MEXICO	e/e/e	j/_/_
62	7812066	BLU SKY	CANADA	g/g/_	c/g/c
63	7812067	OPATA M 85	MEXICO	b/b/b	i/i/i
64	7812068	GLENLEA	CANADA	g/g/g	g/g/c or g
65	7812069	PAVON	MEXICO	b/b/b	h/b or h/b or h
66	7812070	SERI M 82	MEXICO	c/c/c	j/_/_
67	7812071	AC VISTA	CANADA	g/g/g	i/i/i
68	7812072	ANGAS	CANADA	c/c/c	f or g/g/c or g
69	7812073	ATTILA	MEXICO	c/c/c	h/h/h
70	7812074	AVOCET	AUSTRALIA	c/c/c	b/_/b
71	7812075	CARNAMAH	AUSTRALIA	c/c/c	i/i/i
72	7812076	ERNEST	UNITED STATES	d/g/g	d/d/d
73	7812077	HEILO	MEXICO	f/f/f	i/i/i
74	7812078	KATEPWA	CANADA	e/e/e	h/h/h
75	7812079	MARQUIS	CANADA	e/e/_	b'/b/b
76	7812080	MILLEWA	AUSTRALIA	c/c/c	g/g/c or g
77	7812081	NEEPAWA	CANADA	e/e/e	h/h/h
78	7812082	PASTOR	MEXICO	c/c/c	g/g/c or g
79	7812083	PIONEER	CANADA	e/e/e	i/i/i
80	7812084	REBECA F2000	MEXICO	c/c/c	g/g or i/c or g
81	7812085	SPEAR	AUSTRALIA	e/e/e	h/h/h
82	7812086	SPLENDOR	CANADA	e/e/e	f or g /g/c or g
83	7812087	STILETTO	AUSTRALIA	c/c/c	h/h/h
84	7812088	TASMAN	AUSTRALIA	b/b/b	i/i/i
85	7812089	TRIDENT	AUSTRALIA	e/e/e	h/h/h
86	7812090	VERDE	UNITED STATES	f/e/e	h/b/b
87	7812091	WESTONIA	AUSTRALIA	c/c/c	h/h/h
88	7812092	WILGOYNE	AUSTRALIA	d/d/d	h/h/b or h

^a^, the first, second, and third symbol in each column are alleles of Glu-3 loci identified by SDS-PAGE, STS, and SNP markers respectively.

Lines were selected from a set of 103 standard cultivars previously used to establish a uniform classification system of LMW-GS alleles [18,19] and analyzed in various laboratories for the identification of the alleles. The second set (Set2) comprised a selection of 27 Mexican cultivars derived from an overall collection of 150 national cultivars (Table 2). An allele survey using the validated SNP markers was then performed on 54 historical CIMMYT lines (Set 3) representing key germplasm distributed by CIMMYT over the last 50 years (Table 3).

**Table 2:** Compositions of LMW-GS alleles in 27 Mexican wheat cultivars identified by SDS-PAGE, allele specific STS markers and allele specific KASP assays.

No.	Specific Name	Glu-A3*	Glu-B3
1	CORTÁZAR S94	*d/d/d*	*h/h or b/h or b/*
2	SALAMANCA S75	*c/c/c*	*g/g/g*
3	MONARCA F2007	*c/c/c*	*h/h/h*
4	URBINA S2007	*d/d/d*	*h/h/h*
5	JOSECHA F2007	*c/c/c*	*h/h/h*
6	REBECA F2000	*c/c/c*	*g/f/g*
7	TRIUNFO F2004	*d/d/d or f*	*c/g/g*
8	NÁHUATL F2000	*b/c/c*	*i/i/i*
9	DON CARLOS “s”	*c/c/c*	*h/h/h*
10	ROELFS F2007	*b/b/b*	*h/h/h*
11	CACHANILLA F2000	*c/c/c*	*g/g or h/g or h*
12	YECORA F70	*d/d/d*	*h/h/h*
13	NORTEÑA F2007	*d/d/d or f*	*g/h/h*
14	MAYA S2007	*b/b/b*	*h/h/h*
15	SATURNO S86	*c/d/d*	*g/a or g/g*
16	BÁRCENAS S2002	*d/d/d*	*h or b/ h or b /h or b*
17	ALTIPLANO F2007	*c/c/c*	*h or c/h or g/g*
18	NANA F2007	*c/c/c*	*h/h/h*
19	STA. LUCIA “s”	*c/c/c or f*	*h/h/h*
20	TLAXCALA F2000	*c/c/c*	*h/g/g*
21	ONAVAS F2009	*b/b/b*	*h/h/h*
22	VILLA JUAREZ F2009	*c/c/c*	*h/h/h*
23	PALMERIN F2004	*f/f/f*	*b/b/b*
24	RAYON F89	*d/c/c*	*b/b or g/b or g*
25	KRONSTAD F2004	*c/c/c*	*h/h or i/h*
26	ENEIDA F94	*b/b/b*	*h or i/i/i*
27	PROGRESO	*c/c/c*	*b/b/b*

*, the first, second, and third symbol in each column are alleles of *Glu-3* loci identified by SDS-PAGE, STS, and SNP markers respectively.

**Table 3:** Primer sequences for KASP-based assays of SNP markers for LMW-GS alleles.

Primer Name	FAM primer	VIC primer	Common primer	SNP	Vic-Allele	
*Glu-A3a_SNP*	TGGTTGTTGTTGTTGCTGCG	TGGTTGTTGTTGTTGCTGCA	AACAGCAACCACCATTTTCG	A/G	A	*Glu-A3a*
*Glu-A3ac_SNP*	GGAATGTCTTCATTGTGGATTGG	GGAATGTCTTCATTGTGGATTGA	TGGTGCCCGGGCTACTATAA	A/G	A	*Glu-A3ac*
*Glu-A3b_SNP*	CAACAACAACAACAACAACAACAAGTTC	AACAACAACAACAACAACAACAACAAGTTT	GTTGTTGTCGCTGAGCTTGGATGAT	C/T	T	*Glu-A3b*
*Glu-A3d_SNP*	AACAACCCCAACAGTTGGGCCA	AACAACCCCAACAGTTGGGCCT	CGAGTTGCTGCTGCGAYTGTTGTT	A/T	T	*Glu-A3d*
*Glu-A3e_SNP*	ACTAGTTAACACCAATCCACAATGAAG	CACTAGTTAACACCAATCCACAATGAAA	CGAGGAGGGCAMAGACGAGGAA	G/A	A	*Glu-A3e*
*Glu-A3f_SNP*	CCCATGCAAGGTATTCCTCCAG	CCCATGCAAGGTATTCCTCCAA	GCTGCATTGCCACAGGGA	G/A	A	*Glu-A3f*
*Glu-A3g_SNP*	CGACTAAACAACGGTGATCCAACTAT	CGACTAAACAACGGTGATCCAACTAA	GCATCGGAGTTGGTGTCTACTGATA	A/T	T	*Glu-A3g*
*Glu-B3a_SNP*	CGTGTCAATGTGCCGTTGTATAGA	GTGTCAATGTGCCGTTGTATAGG	TGCCAACGCCRAATGGCACACTA	A/G	G	*Glu-B3a*
*Glu-B3b_SNP*	TTACATGTCATGCACTTATCAGGATAT	ACATGTCATGCACTTATCAGGATAC	AAGGTTCCAAAATCRGGTGTAAAAGTGATA	A/G	G	*Glu-B3b*
*Glu-B3c_SNP*	GTCGCAAATGTTGCAGCAGAGC	GGTCGCAAATGTTGCAGCAGAGA	CAACATTGTTGTTGCATCACATGGCAA	C/A	A	*Glu-B3c*
*Glu-B3d_SNP*	CACCATTTTCGCAACAACAACAG	ACCATTTTCGCAACAACAACAT	TGGTATTTGTTGTTGCAGTAGAAC	G/T	T	*Glu-B3d*
*Glu-B3e_SNP*	ATGAAGACCTTCCTCATCTTTGCC	CATGAAGACCTTCCTCATCTTTGCA	GCAATGGCACTTGTCGCCGCAA	C/A	A	*Glu-B3e*
*Glu-B3fg_SNP*	CACAAAGTCTTGCTAGGTCGCAAAT	ACAAAGTCTTGCTAGGTCGCAAAC	CGGCAACATTGTTGCTGCATCACAT	T/C	C	*Glu-B3fg*
*Glu-B3g_SNP*	CTGTTGGGGTTGGGAAACG	CTGTTGGGGTTGGGAAACA	AGCAGCAGCAACCGCAAC	G/A	A	*Glu-B3g*
*Glu-B3h_SNP*	GGTTGTTGTTGTTGGTGGTGCGA	GGTTGTTGTTGTTGGTGGTGCGT	GAGACCATCGCAGCAACAACCATTA	T/A	A	*Glu-B3h*
*Glu-B3i_SNP*	GGTGGTTGTTGTTGCTGCG	GGTGGTTGTTGTTGCTGCA	CAACCATTTCCACAACAGCC	A/G	A	*Glu-B3i*
Glu-Ax1/x2*_SNP^a^	AAGTGTAACTTCTCCGCAACG	ACCTAAGTGTAACTTCTCCGCAACA	CGAAGAAGCTTGGCCTGGATAGTAT	G/A	A	*Ax-null*
*Glu-Ax2_IND*^a^	ATTCTTGTTGTCCTTGTCCTGGCT	CTTGTTGTCCTTGTCCTGGCC	GGTTTCATACTATCCAGGCCAAGCTT	Ins/Del	DEL	Glu-Ax2*
*BX13-1510*^a^	CAACGACCGGGACAAGGGCAAT	CAACGACCGGGACAAGGGCAAC	CTGTGGAGAGGTTGGGTAGTACCC	C/T	C	*Glu-Bx13*
*Glu-D1d_SNP*^a^	ATAGTATGAAACCTGCTGCGGAG	ATAGTATGAAACCTGCTGCGGAC	TACTAAAAAGGTATTACCCAAGTGTAACTT	C/G	G	*Glu-D1d*

^a^ previously published by [22]

### SDS-PAGE analyses

LMW-GS were initially separated by SDS-PAGE in the CIMMYT wheat quality lab based on the extraction method described by [9] with modifications. Concentrations of separation gels were 15.0% T with 1.3% C with a pH of 8.5. Current of running gels was 12.5 mA. The LMW-GS compositions were identified according to [9] and [20] and the gliadins were used as indicators of LMW-GS based on the linkage between LMW-GS and gliadin. The nomenclature system of LMW-GS followed the catalogue of gene symbols for wheat http://wheat.pw.usda.gov/ggpages/awn/53/Textfile/WGC.html.

### DNA extraction and genotyping with STS markers

Genomic DNA was extracted from dried leaves using a modified CTAB (cetyltrimethylammonium bromide) method as described in CIMMYT laboratory protocols [21] and quantified using NanoDrop 8000 spectrophotometer V 2.1.0 (Thermo Fischer Scientific). To avoid inconsistent results derived from using different seed sources or residual heterogeneity within seed lots, the same seed (cut in half) was used for the quality and genetic analyses for Set 1. For Set 2 and 3, leaves from a random bulk of ten seedlings per entry were collected. Functional STS markers published by [16,17] were applied to identify *Glu-A3* and *Glu-B3* alleles in set 1 and set 2. PCR assays in single 10 μl reactions contained final concentrations of 1x Buffer with Green Dye (Promega Corp., US), 200 μM dNTPs, 1.2 mM MgCl_2_, 0.25 μM of each primer, 1U of DNA polymerase (GoTaq®Flexi, Promega Corp., Cat. # M8295) and 50ng of DNA template. The PCR profile was 94°C for 2 min followed by 30 cycles of 94°C for 1 min, 54 to 60°C for 2 min (dependent on the STS primers), 72°C for 2 min. The amplified products were separated on 2–3% agarose gels in TAE buffer.

### SNP marker design and genotyping

KASP assays (LGC Genomics, LCC, Beverly, MA, USA) were developed to genotype the relevant SNPs. Coding sequences of the different *Glu-A3* and *Glu-B3* alleles at each locus were retrieved from the published literature [16,17]. The diagnostic SNPs/InDels for different alleles were identified and KASP primers were developed using the PolyMarker pipeline (http://polymarker.tgac.ac.uk/) following standard KASP guidelines (described in CIMMYT laboratory protocols, [21]). The allele-specific primers were designed carrying the standard FAM (‘5 TGAAGGTGACCAAGTTCATGCT 3′) and HEX (‘5 aGAAGGTCGGAGTCAACGGATT 3’) tails and with the targeted SNP at the 3′ end. A common primer was designed so that the total amplicon length was less than 120 bp. The primer mixture comprised 46 μl ddH2O, 30 μl common primer (100 μM), and 12 μl of each tailed primer (100 μM). Assays were tested in 384-well formats and set up as 4 μl reactions containing 2.5ml water, 2.5 ml 2×KASP Reaction mix, 0.07 ml Assay mix and 50ng of dried DNA with a PCR profile of 94°C for 15 min activation time followed by 20 cycles of 94°C for 10 sec, 57°C for 5 sec, 72°C for 10 sec and followed by 18 cycles of 94°C for 10 sec, 57°C for 20 sec, and 72°C for 40 sec. Fluorescence was read as an end point reading (PHERAstar FSX, BMG Labtech) at 25°C.

## Results

### SNP marker validation

#### Segregation of LMW-GS using SDS-PAGE

**Fig 1:**
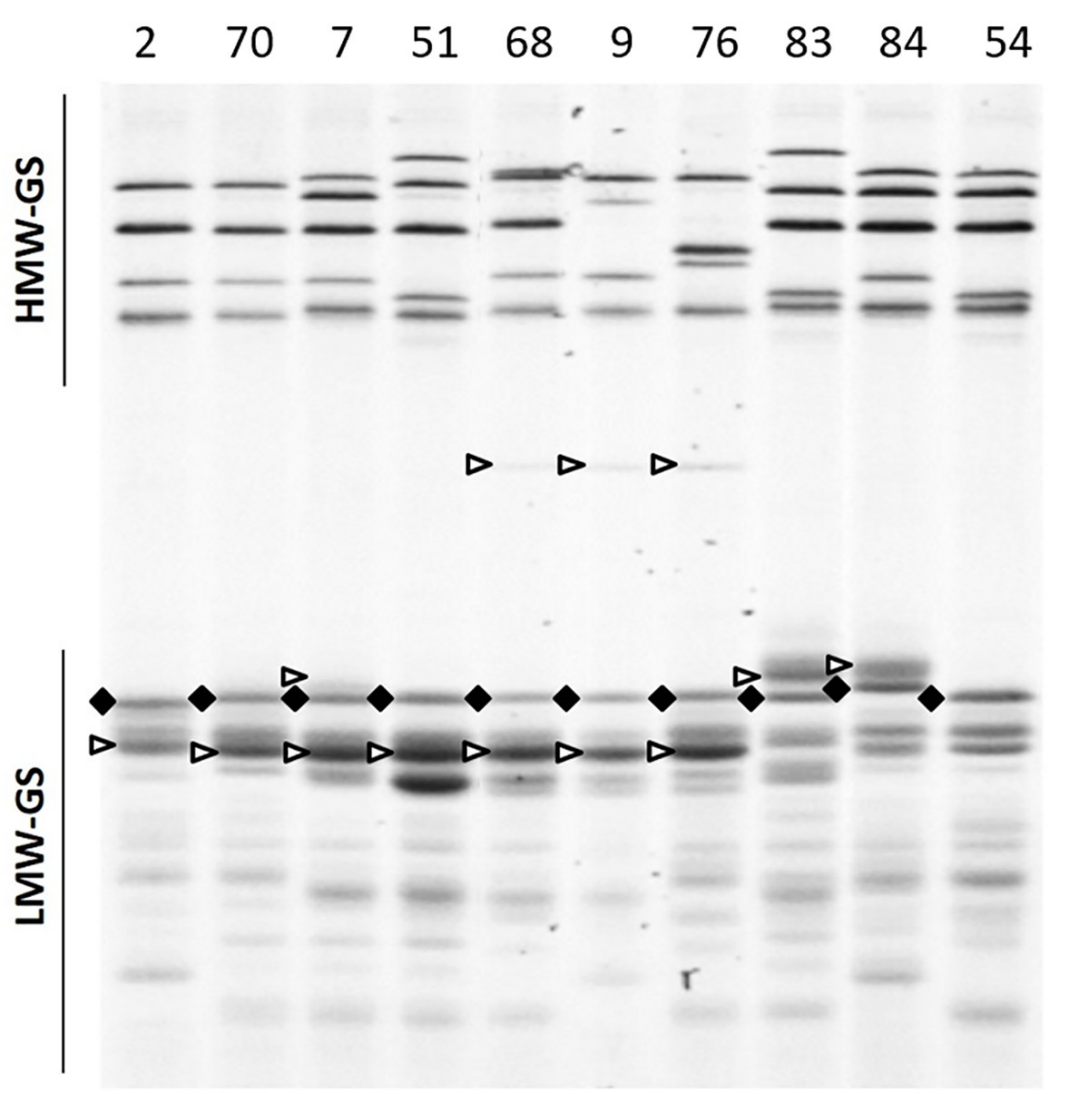
High and low-molecular-weight subunit composition (HMW- and LMW-GS) by SDS-PAGE. Glu-A3 alleles are indicated by a rhombus, Glu-B3 alleles by an arrow. Labels correspond to the entry numbers in Table 1.

The LMW-GS compositions identified by SDS-PAGE are listed in Tables 1 and 2. At the *Glu-A3* locus, alleles *Glu-A3b* and *Glu-A3e* could be easily detected. Allele *Glu-A3c*, *Glu-A3d* and *Glu-A3f* were more difficult to separate [17] (Fig 1). At the *Glu-B3* locus, the alleles *Glu-B3d*, *Glu-B3h* and *Glu-B3i*, each carried the slowest LMW-GS bands in SDS-PAGE. As previously reported, allele *Glu-B3f* could not always be reliably discriminated from *Glu-B3g* since these bands had very similar mobilities. This was the case for six lines in Set 1. In four lines in Set 2, the allele *Glu-B3h* was detected together with alleles *Glu-B3b*, *c or i*.

#### Verification of LMW-GS via allele specific STS markers

**Fig 2:**
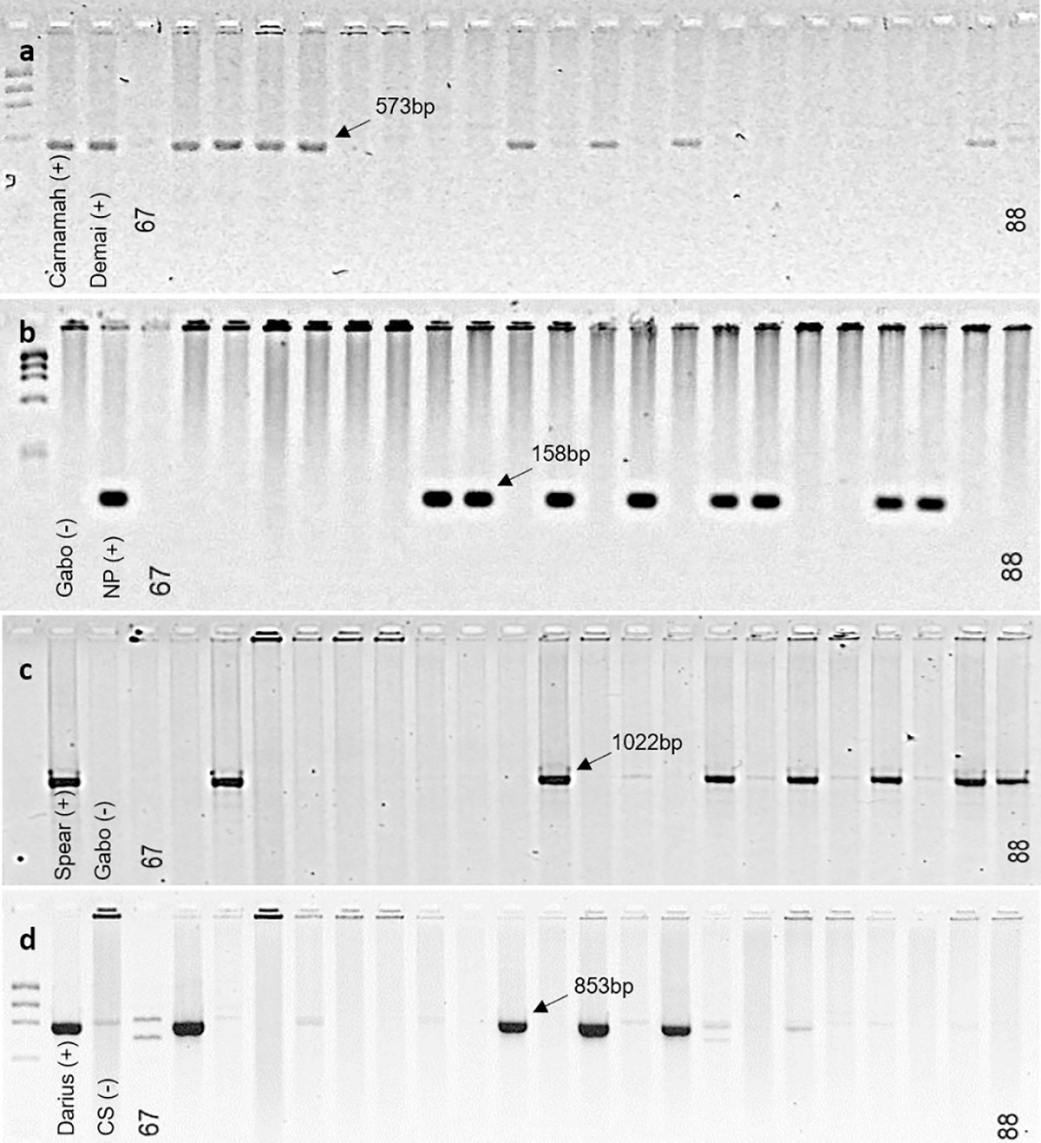
Electrophoresis of PCR products on agarose gels using allele-specific STS markers, which positively effect quality. a KASP assay for *Glu-A3ac*, b KASP assay for *Glu-A3e*, c KASP assay for *Glu-B3h*, d KASP assay for *Glu-B3g*. DNA ladder PhiX174. Labels belong to respective checks and entry numbers in Table 1.

Seven primer pairs were used to identify *Glu-A3* alleles and nine primer pairs for *Glu-B3* alleles. The amplified fragments were of the same size than previously published in [16,17] (Fig 2). The *Glu-A3* and *Glu-B3* alleles could be differentiated well, however, the STS markers could not identify allele *Glu-B3j*, which resulted in 16 missing values for the 176 data points. Overall 78.3% of the detected alleles were identical between STS markers and SDS-PAGE. In 10.9% STS markers showed the same allele than SDS-PAGE plus an additional allele, in 11.5% the allele was different. In Set 1, different *Glu-A3* alleles were identified for the lines CAPPELLE DESPREZ (*Glu-A3c* instead of *Glu-A3d*), ERNEST (*Glu-A3g* instead of *Glu-A3d*), GREEBE (*Glu-A3e* instead of *Glu-A3c*), JING 411 (*Glu-A3d* instead of *Glu-A3c*), KLEIN PROTEO (*Glu-A3c* instead of *Glu-A3g*), NIDERA BAGUETTE 20 (*Glu-A3c* instead of *Glu-A3f*), and VERDE (*Glu-A3e* instead of *Glu-A3f*) when compared to the SDS-PAGE results (Table 1). For *Glu-B3*, detected alleles differed for the lines BLU SKY (*Glu-B3g* instead of *Glu-B3c*), COURTOT (*Glu-B3f* instead of *Glu-B3b*), JING 411 (*Glu-B3d* instead of *Glu-B3h*), NORIN 61 (*Glu-B3fg* instead of *Glu-B3i*), ORCA (*Glu-B3g* instead of *Glu-B3d*), SALOMNE (*Glu-B3g* instead of *Glu-B3c*), SHINCHUNAGA (*Glu-B3fg* instead of *Glu-B3i*), THESEE (*Glu-B3h* instead of *Glu-B3g*), and VERDE (*Glu-B3b* instead of *Glu-B3h*). In Set 2, different *Glu-A1* alleles were observed for the three lines, NÁHUATL F2000 (*Glu-A3c* instead of *Glu-A3b*), SATURNO S86 (*Glu-A3d* instead of *Glu-A3c*) and RAYON F89 (*Glu-A3c* instead of *Glu-A3d*) in comparison to the SDS-PAGE results, while different *Glu-B3* alleles were detected for four out of the 27 lines, namely REBECA F2000 (*Glu-B3f* instead of *Glu-B3g*), TRIUNFO F2004 (*Glu-B3g* instead of *Glu-B3c*), NORTEÑA F2007 (*Glu-B3h* instead of *Glu-B3g*), TLAXCALA F2000 (*Glu-B3g* instead of *Glu-B3h*).

#### Validation of LMW-GS using the newly designed KASP assays

**Fig 3:**
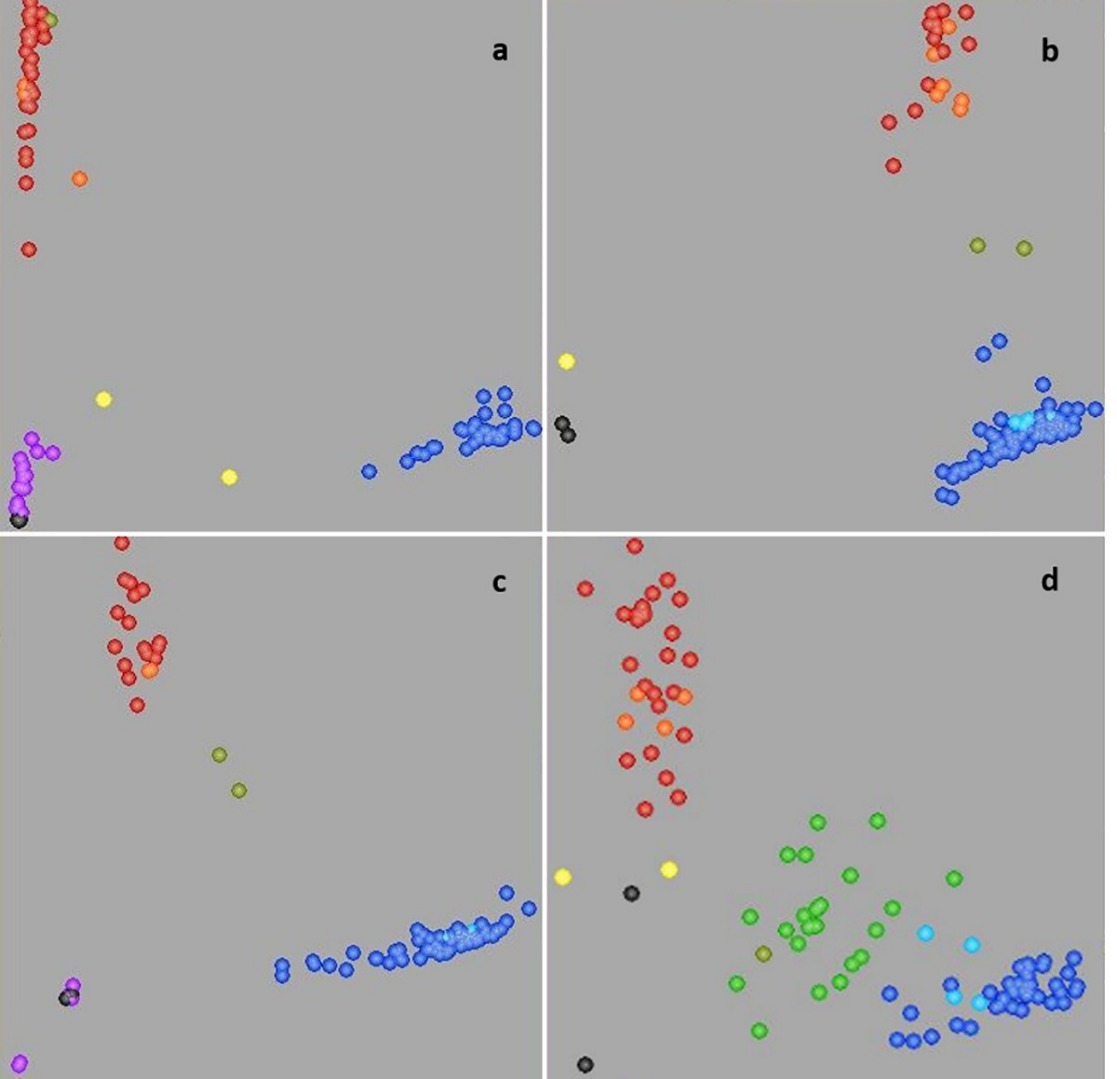
Scatter plots for selected KASP assays which positively effect quality showing clustering of varieties on the *X*- (FAM) and *Y*- (HEX) axes. Varieties colored *blue* have the FAM-type allele; varieties colored *red* have the VIC-type allele; *black dots* represent the NTC (non-template control). a KASP assay for *Glu-A3ac*, b KASP assay for *Glu-A3e*, c KASP assay for *Glu-B3h*, d KASP assay for *Glu-B3g*.

KASP assays designed are presented in Table 3 and the assays are aligned to their respective allele sequences in S1 File. All assays formed clear sample clusters with the allele labeled with the fluorescence ‘VIC’ representing the designated *Glu-A3* and *Glu-B3* allele (Fig 3). Similar to the STS markers, a KASP assay for the *Glu-B3j* allele was not designed, leading to 20 missing values for 176 data points. Among Set 1 and Set 2, 77.7% of the data points were identical between the KASP assays and SDS-PAGE. In 12.6% of the lines, the KASP assays showed the same allele as SDS-PAGE plus an additional allele. This was mainly due to *Glu-B3c* and *Glu-B3g* alleles simultaneously detected with the KASP. In 10.0% of the lines, alleles identified by KASP were different from alleles by SDS-PAGE. A higher percentage (83.6%) of alleles was identical and a lower percent of alleles (1.9%) differed between the KASP assays and the STS markers, while in 14.5% cases one or the other marker type showed more than one allele. In Set 1, parallel to the STS markers, different *Glu-A3* alleles were identified for the lines ERNEST (*Glu-A3g* instead of *Glu-A3d*), GREEBE (*Glu-A3e* instead of *Glu-A3c*), CAPPELLE DESPREZ (*Glu-A3c* instead of *Glu-A3d*), JING 411 (*Glu-A3d* instead of *Glu-A3c*) and VERDE (*Glu-A3e* instead of *Glu-A3f*) when compared to SDS-PAGE (Table 1). The KASP assay disagreed with the reading of the STS marker but agreed with SDS-PAGE for the line NIDERA BAGUETTE 20 (*Glu-A3f*). At the *Glu-B3* locus, similarly to the STS marker, lines COURTOT (*Glu-B3f* instead of *Glu-B3b*), NORIN 61 (*Glu-B3fg* instead of *Glu-B3i*), ORCA (*Glu-B3g* instead of *Glu-B3d*), SHINCHUNAGA (*Glu-B3fg* instead of *Glu-B3i*), THESEE (*Glu-B3h* instead of *Glu-B3g*) and VERDE (*Glu-B3b* instead of *Glu-B3h*) showed a different allele compared to SDS-PAGE. However, the KASP assay confirmed the allele of SDS-PAGE for lines BLU SKY (*Glu-B3c*) and JING 411 (*Glu-B3h*). For Set 2, the KASP assays revealed the same results as the STS markers for lines TRIUNFI F2004, NÁHUATL F2000, NORTEÑA F2007, SATURNO S86, TLAXCALA F2000 and RAYON F89. The results were, however, different for the line REBECA F2000 and ANTIPLANO F2007.

### Allele survey in CIMMYT historical lines

The newly designed and validated SNP markers were subsequently applied to survey the *Glu-3* alleles in set 3 including 54 CIMMYT historical lines. Furthermore, SNP markers previously developed for *Glu-A1*, *Glu-B1* and *Glu-D1* loci were deployed (Table 4) to determine the HMW-GS in the same set of lines. At the *Glu-A1* locus, 72% of the lines carried the Ax2* subunit and 28% the Ax1 subunit (Table 3). None of the line carried the null allele causing a low-quality score. Subunit Bx13 at the *Glu-B1* locus was the only allele to be amplified with SNP markers. KASP assays for the subunit Bx7OE exist [22] but were not found to provide reliable results based on a lack of clustering in this study. Among the CIMMYT historical lines two lines (OPATA M85 and WHEAR/SOKOLL) carried this allele. For *Glu-D1*, the subunit combination of Dx5 and Dy10 was found in 75% of the lines, while the allelic pair Dx2 + Dy12 with negative effects on bread making quality was found in 25% of the lines including six lines released after the year 2000. The *Glu-A3* locus showed lower allelic diversity than *Glu-B3* locus. Five different alleles were found at the *Glu-A3* locus. The most frequent *Glu-A3* allele was allele c, present in 75% of the lines, followed by allele b present in 15% of the lines. One to three lines carried alleles d, e, and f (Table 3). Seven different alleles were found at the *Glu-B3* locus. Thirty-four percent of the lines carried *Glu-B3* allele h, while 23% and 17% of the lines carried allele g and b, respectively. Alleles d, f, i and c, were observed in less than 10% frequency. The 54 lines were released by CIMMYT during the last 50 years. No obvious trend in the distribution of *Glu-3* alleles was evident across years.

**Table 4:** Compositions of HMW and LMW-GS alleles in 54 CIMMYT wheat cultivars identified by allele specific KASP assays.

			SNP marker				
No.	Cultivar	Year	*Glu-A1*^*a*^	*Glu-B1*^*a*^	*Glu-D1*^*a*^	*Glu-A3*	*Glu-B3*
	**Pedigree**						
1	SIETE CERROS T66	1966	2*		*x2+y12*	*b*	*g*
2	SONALIKA	1967	2*		*2+12*	*c*	*i*
3	PAVON F 76	1976	2*		*5+10*	*b*	*h*
4	WL 711	1979	*1*		*_*	*c*	*g/c*
5	LOK 1	1981	2*		*2+12*	*c*	*d*
6	SERI M 82	1982	*1*		*5+10*	*c*	*g*
7	HUW 234	1984	2*		*2+12*	*c*	*d*
8	OPATA M 85	1985	2*	*13*	*2+12*	*b*	*i*
9	ATTILA	1990	2*		*5+10*	*c*	*h*
10	PBW343	1990	*1*		*5+10*	*c*	*g*
11	INQALAB 91	1991	2*		*2+12*	*c*	*f*
12	BAVIACORA M 92	1992	2*		*5+10*	*c*	*h*
13	PFAU/WEAVER	1995	*1*		*5+10*	*c*	*f/c*
14	SUPER SERI #1	1999	*1*		*5+10*	*c*	*h*
15	WEEBILL1	1999	2*		*5+10*	*c*	*h*
16	TACUPETO F2001	2001	2*		*5+10*	*b*	*h*
17	VOROBEY	2002	*1*		*2+12*	*c*	*f*
18	ATTILA*2/PBW65	2003	2*		*2+12*	*c/d*	*i*
19	PRL/2*PASTOR	2004	*1*		*5+10*	*d*	*g*
20	PBW65/2*PASTOR	2004	*1*		*5+10*	*d*	*g*
21	SW89.5277/BORL95//SKAUZ/3/PRL/2*PASTOR/4/HEILO	2004	*1*		*5+10*	*d*	*_*
22	SEHER 06	2006	2*		*5+10*	*c*	*b*
23	HD2687	2006	2*		*2+12*	*e*	*d*
24	NAVOJOA M2007	2007	2*		*5+10*	*c*	*h*
25	ROELFS F2007	2007	2*		*5+10*	*b*	*h*
26	MISR 1	2007	*1*		*5+10*	*c*	*g*
27	LASANI-08	2008	2*		*5+10*	*b*	*i*
28	MUNAL #1	2008	2*		*5+10*	*c*	*b*
29	QUAIU #1	2008	2*		*5+10*	*c*	*h*
30	DANPHE #1	2009	*1*		*5+10*	*c*	*i*
31	BABAX/LR42//BABAX*2/4/SNI/TRAP#1/3/KAUZ*2/TRAP//KAUZ	2008	2*		*5+10*	*c*	*h*
32	GRACKLE #1	2008	2*		*5+10*	*c*	*b*
33	ELVIRA/5/CNDO/R143//ENTE/MEXI75/3/AE.SQ/4/2*OCI	2008	2*		*5+10*	*c*	*h*
34	FRANCOLIN #1	2008	2*		*5+10*	*c*	*b*
35	NELOKI	2009	2*		*5+10*	*c*	*g/c*
36	BAJ #1	2010	2*		*5+10*	*b*	*b*
37	KACHU #1	2010	2*		*5+10*	*b*	*b*
38	BL3063/VIJAY	2010	2*		*2+12*	*c*	*d*
39	TRCH*2/3/C80.1/3*QT4118//3*PASTOR	2011	*1*		*5+10*	*c*	*g*
40	WHEAR/KRONSTAD F2004	2011	*1*		*5+10*	*c*	*f*
41	CNDO/R143//ENTE/MEXI_2/3/AEGILOPS SQUARROSA (TAUS)/4/WEAVER/5/PICUS/6/TROST/7/TACUPETO F2001	2011	2*		*5+10*	*c*	*h*
42	SHA7/VEE#5/5/VEE#8//JUP/BJY/3/F3.71/TRM/4/2*WEAVER/6/SKAUZ/PARUS//PARUS	2011	2*		*5+10*	*c*	*g*
43	WHEAR/SOKOLL	2011	*1*	*13*	*2+12*	*c*	*b*
44	HUW234+LR34/PRINIA*2//KIRITATI	2012	*1*		*2+12*	*f*	*d*
45	KACHU/SAUAL	2012	2*		*5+10*	*c*	*b*
46	FRET2*2/4/SNI/TRAP#1/3/KAUZ*2/TRAP//KAUZ/5/PARUS/6/FRET2*2/KUKUNA	2012	2*		*2+12*	*c*	*h*
47	PFAU/SERI.1B//AMAD/3/WAXWING	2012	2*		*5+10*	*c*	*g*
48	ATTILA*2/PBW65/6/PVN//CAR422/ANA/5/BOW/CROW//BUC/PVN/3/YR/4/TRAP#1/7/ATTILA/2*PASTOR	2012	2*		*5+10*	*c*	*h*
49	PBW343*2/KUKUNA//PARUS/3/PBW343*2/KUKUNA	2012	2*		*5+10*	*c*	*b*
50	REEDLING #1	2012	2*		*5+10*	*c*	*h*
51	BECARD/QUAIU #1	2014	2*		*5+10*	*c*	*h*
52	BABAX/LR42//BABAX*2/3/KUKUNA/4/CROSBILL #1/5/BECARD	2014	2*		*5+10*	*c*	*h*
53	KACHU//WBLL1*2/BRAMBLING	2014	2*		*5+10*	*c*	*h*
54	PFAU/SERI.1B//AMAD/3/WAXWING/4/BAJ #1	2014	2*	* *	*5+10*	*c*	*g*

^a^ previously published by [22]

## Discussion

To determine wheat quality, several measurements of wheat grain, flour, dough, and final products must be assessed within breeding programs [23]. Several of the measurements are greatly limited by the amount of seed required and the cost and time of testing. Grain tests can be done on a small scale, quickly and cost effectively, making high-throughput implementation possible. However, dough rheology and end-use tests require large quantities of grain, which is destructed for milling into flour, limiting their implementation to advanced stages in a breeding pipeline [24]. Therefore, marker assisted selection (MAS) could be beneficial for these traits.

Gluten is the most important factor in determining bread making quality and is largely optimized by deployment of appropriate HMW-GS and LMW-GS alleles [25]. DNA markers linked to most of the individual gluten subunits or alleles exist but still require gel electrophoresis while higher-throughput and cost-effective genotyping platforms are available. The newly designed SNP markers for *Glu-A3* and *Glu-B3* alleles in this study were validated by comparing results to those derived by SDS-PAGE and STS marker methods, while the former was taken the standard. Various laboratories have tried to unify the nomenclature system of LMW-GS based upon the relative mobilities in SDS-PAGE and identified the protein composition of 103 reference lines, of which 75 were included in Set 1 of our study [18,19]. The correlation between the three methods in this study was relatively high. The SNP markers correlated slightly better with SDS-PAGE than the STS markers (10% vs. 11.5% of disagreement) but showed overall a somewhat higher number of multiple alleles (12.6% vs. 10.9%). We expect that the disagreement in allele detection has several reasons. Each method has a different discrimination power. Several of the alleles inconsistent across methods included SDS-PAGE bands that were difficult to separate (e.g., *Glu-A3c* and *d*), thus might not have been correctly identified. In addition, SDS-PAGE does not allow to read eventual residual heterozygote loci. The *Glu-3* STS markers are dominant markers and can therefore produce false negatives, which resulted in more missing data. Similar to SDS-PAGE, heterozygote loci cannot be detected. Targeting only a single SNP and not a larger gene region, the KASP assays can be less allele specific. A larger number of multiple alleles were derived from the two KASP assays, mainly for the alleles *Glu-B3c* and *Glu-B3g*, indicating some lack of allele specificy. However, KASP assays, however, can detect heterozygote loci. The disagreement of alleles in Set 2 might have also be derived from the different seed lots used for the SDS-PAGE and molecular marker analyses. Residual heterozygosity within and between the seed lots is not as uncommon. Falling in the category of a high-throughput genotyping platform the KASP assays show some extra benefits in comparisons to SDS-PAGE and/or STS markers as 1) early breeding generation segregating materials can be evaluated, due to their co-dominant inheritance; 2) production costs are reduced; and 3) highly automated options are available that require less time to generate the results. The advantage of co-dominant markers in MAS strategies is that they allow a more direct assessment of the frequencies of the target alleles and thus progeny testing of selected individuals at later generations (e.g., F5 or F6) to recover homozygotes is not required. The cost for evaluating a line or sample with SDS-PAGE is around 30 USD at CIMMYT. Using a multi-gel buffer chamber allows running both gliadin and glutenin extracts at the same time providing reliable results, but not all alleles can be easily distinguished and results across different laboratories vary significantly. The sample cost using DNA markers consist of a DNA extraction cost and the genotyping cost. Genotyping using KASP is cheaper than using STS markers, especially when many samples are evaluated. In this study, 21 KASP assays were used to evaluate 54 historical CIMMYT lines for *Glu-1* and *Glu-3* alleles. Current genotyping cost per sample using 21 KASP assay with CIMMYT service providers would vary between 2.65 to 6.00 USD depending on the overall number of samples to be evaluated (1536 and 384 formats, respectively). However, while the cost of KASP assays are significantly less, not all HMW-GS and LMW-GS alleles can be identified yet (e.g., *Glu-B1* and *Glu-D3* alleles). Thus, using the currently available KASP assays alone will not provide the full composition of alleles, but KASP are ideal to be used in MAS when certain allele combinations are to be selected. Attempts to develop markers for other *Glu-B1*, *Glu-D1* and *Glu-D3* alleles are ongoing [26]. At CIMMYT with a very well-established wheat quality laboratory the designed KASP assays presented in this study are currently used to verify uncertain SDS-PAGE results during the evaluation of advanced breeding lines in preliminary yield trails. KASP assays associated with *Glu-1* alleles are used in MAS in early breeding generations. For breeding programs with limited resources or limited access to a wheat quality laboratory, the SNP markers present a great alternative tool for screening Glutenin protein composition.

[19] reported the composition of LMW-GS alleles of 103 wheat cultivars including 75 of the 88 cultivars in our Set 1. Almost all our SDS-PAGE results were similar to the study of [19] with only a few exceptions (four out of 75). E.g., line AC VISTA in our study showed allele *Glu-A3g*, while in [19] allele *Glu-A3e*, line BLUE SKY in our study showed allele *Glu-B3c*, while in [19] *Glu-B3g*. For the overall eight lines with different allele scores between SDS-PAGE and both DNA marker types (STS and KASP), five lines were also genotyped with STS markers in the same study of [19]. In four cases, the STS marker results matched the SDS-PAGE results in [19], while in one case DNA marker results were the same in both studies, thus the markers reporting a clearly different allele than SDS-PAGE. One reason for any discrepancy between the two studies could be the use of a different seed source or residual heterogeneity in the lines.

Finally, 54 historical CIMMYT lines were evaluated with available *Glu-1* and *Glu-3* KASP assays. At the *Glu-1* loci, the frequency of the subunits Ax1 or Ax2* and Dx5 + Dy10 were high. These subunits are positively associated with bread making quality [11,27–31]. [32] carried out an extensive survey of LMW-GS in common wheat cultivars by SDS-PAGE and detected 20 banding patterns. Subsequently, six protein alleles were found for the *Glu-A3* locus (a, b, c, d, e, f), nine for the *Glu-B3* (a, b, c, d, e, f, g, h, i). With respect to effects on dough quality, various *Glu-3* alleles were ranked for Rmax (maximum dough resistance, an indicator of dough strength), and the rankings of alleles were b > d > e > c at the *Glu-A3* locus, i > b = a > e = f = g = h > c at *Glu-B3* [29,33,34]. [35] found that the composition bbb for *Glu-A3*, *Glu-B3* and *Glu-D3*, respectively, gave the best extensibility, and the composition bbc was almost as extensible. [36] considered allele d at *Glu-A3* locus, allele b at *Glu-B3* locus had greater quality parameters than their counterpart alleles. The most frequent *Glu-A3* allele in the CIMMYT historical lines was allele *Glu-A3c* (76%), followed by allele *Glu-A3b* (15%). This result was consistent with the study by [37], who analyzed 273 CIMMYT lines with SDS-PAGE. The same authors found overall four different *Glu-A3* alleles. In this study we found the same four *Glu-A3* alleles, and additionally allele *Glu-A3f* in line HUW234+LR34/PRINIA*2//KIRITATI. Alleles *Glu-A3e* in hexaploid wheat being a null allele was found in only one line (HD2687). The most frequent *Glu-B3* allele in same set of 54 CIMMYT historical lines was allele *Glu-B3h* (34.0%) followed by allele *Glu-B3g* (22.6%) and allele *Glu-B3b* (17.0%). These results were similarly consistent with the study of [37] in which however, 23% percent of the CIMMYT lines were also reported to carry the allele *Glu-B3j*. This allele is associated with the 1B.1R translocation and exhibits a strongly negative effect on all quality parameters. Despite some intends, a reliable SNP marker for the *Glu-B3j* has not developed yet. The *Glu-B3j* allele was therefore not detected in lines known to carry the 1B.1R translocation such as Seri M82. Overall, while the frequencies of HMW-GS positively associated with bread making quality are high in CIMMYT wheat varieties, there is the opportunity to improve the deployment of LMW-GS alleles.

## Supporting information

S1 File Sequence alignment of designed KASP assays associated to *Glu-A3* and *Glu-B3* alleles. All 200bp sequences are derived from the S1 File in the publications of Wang et al. 2009 and 2010. The target SNP in highlighted in brackets, the KASP allele-specific primers and common primer are underlined.
(DOC)
