# Supplementary material for: SNP markers for low molecular glutenin subunits (LMW-GSs) at the Glu-A3 and Glu-B3 loci in bread wheat
Source: PLoS One. 2020 May 12;15(5):e0233056. doi: 10.1371/journal.pone.0233056 (PMC7217469; doi:10.1371/journal.pone.0233056)
Supplement: S1 File — All 200bp sequences are derived from the S1 File in the publications of Wang et al. 2009 and 2010. The target SNP in highlighted in brackets, the KASP allele-specific primers and common primer are underlined. (DOC) [file pone.0233056.s001.doc]

**Supplementary File 1.** Sequence alignment of designed KASP assays associated to *Glu-A3* and *Glu-B3* alleles. All 200bp sequences are derived from the supplementary materials in the publications of Wang et al. 2009 and 2010. The target SNP in highlighted in brackets, the KASP allele-specific primers and common primer are underlined.

**Glu-A3a_SNP**

T: Glu-A3a

C: non Glu-A3a

CCATATTCGCAGCAACAACAGCCACCATATTCGCAGCAACAACAACCACCATTTTCGCAGCAACAGCAACCACCATTTTCGCAGCAACAACAACCACCATTTT**[T/C]**GCAGCAACAACAACAACCACCATTTACACAGCAACAACAACCAYCGTTTTCACAACAGCCACCAATTTCACAGCAGCAGCAGCAGCAACAAC

**--------------------------------------------------------**

**Glu-A3ac_SNP**

C: Glu-A3ac

T: non Glu-A3ac

TGCAAAGAAGAAAAGAGGTGGTGCCCGGGCTACTATAAATAGGMMTGAAGTATAAAGATCATCACAAGCACAAGCATCAAAACCAAGCAACACTAGTTRACA[T/C]CAATCCACAATGARGACMTTCCTYGTCTTTKCCCTCCTCGCTCTTGCGGCRGCAAGTGYSGTTGYGCAAATTTCACAGCAACAACAACAACAA

**--------------------------------------------------------**

**Glu-A3b_SNP**

T: Glu-A3b

C: non Glu-A3b

TGAGTTAATCCGTGCTATCATCTACTCTATCATCCTGCAGCAACAACAACAACAACAACAACAACAAGTT**[T/C]**AGAGTATCATCCAAGCTCAGCGACAACAACCCCAACAGTTGGGCCAATGTGTCTCCCAACCCCAACAACAATCGCAGCAGCAACTCGGGCAACAACCTCAACAACAACAATTGGCACAGGGTACC

**--------------------------------------------------------**

**Glu-A3d_SNP**

T: Glu-A3d

A: non Glu-A3d

CAACAACAACAACAAcaacaacaacaacaacaagaacaacaacaacaacaacaacaacatcaacaaGTTCAGAGTATCATCCAAGCTCAGCAACAACAACCCCAACAGTTGGGCC**[T/A]**ATGTGTCTCCCAACCCCAACAACARTCGCAGCAGCAACTCGGGCAACAACCTCAACAACAACAATTGGCACAGGGTACCT

**--------------------------------------------------------**

**Glu-A3e_SNP**

A: Glu-A3e

G: non Glu-A3e

AAGCCGGTGCAAAGAAGGAAAAGAGGTGGTGTCCCGGCAACTATAAATAGGCATGAAGTATAAAGATCATCACAAGCACAAGCATCAAAGCCAAACAACACTAGTTAACACCAATCCACAATGAA**[A/G]**ACCTTCCTCGTCTKTGCCCTCCTCGCTCTTGCGGCGGCAAGTGCCGTTGCGCAAATTTCACAGCAACAAC

**--------------------------------------------------------**

**Glu-A3f_SNP**

G: Glu-A3f

A: non Glu-A3f

AGCTAAACCCATGCAWGGTATTCCTCCA**[G/A]**CAGCAGTGCATCCCTGTGGCAATGCAGCGATGTCTTGCTAGGTCACAAATGTTGCAGCAGAGCATTTGCCATGTGATGCAGCRACAATGTTGCCAGCARTTGCGGCAAATCCCCGAGCAATCCCGCCATGAGTCAATCCGTGCTATC

**--------------------------------------------------------**

**Glu-A3g_SNP**

T: Glu-A3g

A: non Glu-A3g

ATGTGCCGTTGTACGAAACCACCACTAGTGTGCCATTAGGCGTTGGCATCGGAGTTGGTGTCTACTGATAAGAAAAGATCTCTAGTAATAT**[T/A]**TAGTTGGATCACCGTTGTTTAGTCGATGGATATGTCGATGTAGCGGTGACAAATAAAGTGTCACACAACGTCATGTGTGACCCACTCAAACTAGTTGTTTAAAT

**--------------------------------------------------------**

**Glu-B3a_SNP**

G: Glu-B3a

A: non Glu-B3a

TTTTGCAGCCACACCAGATAGCTCAGCTTGAGGTGATGACTTCCATTGCGCTCCGTACCCTGCCAATGATGTGCCGTGTCAATGTGCCGTTGTATAG**[G/A]**ACCACCACTAGTGTGCCATTYGGCGTTGGCACTGGAGTTGGTGCCTACTGATAAGGAAAGGTCTCTAGTAATATATAGTTGGATCACCGTTTCTTAGT

**--------------------------------------------------------**

**Glu-B3b_SNP**

G: Glu-B3b

A: non Glu-B3b

CACCCTATACAAGGTTCCAAAATCRGGTGTAAAAGTGATA**[G/A]**TATCCTGATAAGTGCATGACATGTAAAGCGAATAAGGCAAGTTATCTATAACAAAGATTATGTACTTTTTCCCAAGTCGGGTGTAAAAGTGATACTATCCTGATAAATGCGTGACATGTAAAGTGAATAAGGCAAGTCATCTACTTCAAACATCA

**--------------------------------------------------------**

**Glu-B3c_SNP**

A: Glu-B3c

C: non Glu-B3c

GCAAGGTATTCCTCCAGCAGCAATGCAGCCCTGTGGCTATGCCACAAAGTCTTGCTAGGTCGCAAATGTTGCAGCAGAG**[A/C]**AGTTGCCATGTGATGCAACAACAATGTTGCCAGCAGTTGCCGCAAATCCCCCAGCAATCCCGCTATGAGGCAATCCGTGCTATCGTCTACTCCATCATCCTGCAAGAACAACAACA

**--------------------------------------------------------**

**Glu-B3d_SNP**

T: Glu-B3d

G: non Glu-B3d

ACAACCACCATTTTCGCAACAACAACAACAACCAATTCTACCGCAACAACCACCATTTTCGCAACAACAACA**[G/T]**CCAGTTCTACTGCAACAACAAATACCATTTGTTCATCCATCTATCTTGCAGCAACTAAACCCATGCAAGGTATTCCTCCAGCAGCAATGCAGCCCTGTGGCTATGCCACAAAGTCTTGCTAGG

**--------------------------------------------------------**

**Glu-B3e_SNP**

A: Glu-B3e

C: non Glu-B3e

GCACAAGCATCAAAACCAAGAAATACTAGTTAACACTAGTCCACCATGAAGACCTTCCTCATCTTTGC**[A/C]**CTCCTCGCCGTTGCGGCGACAAGTGCCATTGCACAAATGGAGAATAGCCACATCCCTGGTTTGGAGAGACCATCGCAGCAACAACCATTACCACCACAACAAACATTATCGCACCAACAACCACAAC

**--------------------------------------------------------**

**Glu-B3fg_SNP**

C: Glu-B3fg

T: non Glu-B3fg

GCAGCTAAACCCATGCAAGGTATTCCTCCAGCAGCAATGCAGCCCTGTGGCAATGCCACAAAGTCTTGCTAGGTCGCAAA**[C/T]**GTTGTGGCAGAGTAGTTGCCATGTGATGCAGCAACAATGTTGCCGGCAGCTGCCGCAAATCCCCGAACAATCACGCTAYGATGCAATCCGTGCCATCATCTACTCGATCGTCCTA

**--------------------------------------------------------**

**Glu-B3g_SNP**

T: Glu-B3g

C: non Glu-B3g

TCATCTACTCCATCGTCCTGCAAGAACAACAACAGGTTCGGGGTTCCATCCAAACTCAGCAGCAGCAACCGCAACAGTTGGGCCAATG**[C/T]**GTTTCCCAACCCCAACAGCAGTCACAGCAGCAACTCGGGCAACAACCTCAACAACAACAATTGGCACAGGGTACCTTTTTGCAGCCACACCAGATAGCTCAGCTTGA

**--------------------------------------------------------**

**Glu-B3h_SNP**

A: Glu-B3h

T: non Glu-B3h

GGCGACAAGTGCCATTGCACAAATGGAGAATAGCCACATCCCTGGTTTGGAGAGACCATCGCAGCAACAACCATTACCACCACAACAAACATTA**[A/T]**CGCACCACCAACAACAACAACCCATCCAACAACAACCACACCAATTTCCACAACAGCAACCATGTTCACAGCAACAACAACAACCACCATTATCGCAACAA

**--------------------------------------------------------**

**Glu-B3i_SNP**

A: Glu-B3i

C: non Glu-B3i

TACCACTACAACAAATATTATGGTACCAACAACAACAACCCATCCAACAACAACCACAACCATTTCCACAACAGCCACCATGTTCACAGCAACAACAACCACCATTAT**[T/C]**GCAGCAACAACAACCACCATTTTCACAACAA
